# Specification of murine ground state pluripotent stem cells to regional neuronal populations

**DOI:** 10.1038/s41598-017-16248-x

**Published:** 2017-11-22

**Authors:** Walaa F. Alsanie, Jonathan C. Niclis, Cameron P. Hunt, Isabelle R. De Luzy, Vanessa Penna, Christopher R. Bye, Colin W. Pouton, John Haynes, Jaber Firas, Lachlan H. Thompson, Clare L. Parish

**Affiliations:** 1 The Florey Institute of Neuroscience and Mental Health, The University of Melbourne, Melbourne, Australia; 2 Monash Institute of Pharmaceutical Sciences, Monash University, Melbourne, Australia; 3 The Australian Regenerative Medicine Institute, Monash University, Melbourne, Australia; 4 Present Address: The Department of Medical Laboratories, The Faculty of Applied Medical Sciences, Taif University, Taif, Saudi Arabia

## Abstract

Pluripotent stem cells (PSCs) are a valuable tool for interrogating development, disease modelling, drug discovery and transplantation. Despite the burgeoned capability to fate restrict human PSCs to specific neural lineages, comparative protocols for mouse PSCs have not similarly advanced. Mouse protocols fail to recapitulate neural development, consequently yielding highly heterogeneous populations, yet mouse PSCs remain a valuable scientific tool as differentiation is rapid, cost effective and an extensive repertoire of transgenic lines provides an invaluable resource for understanding biology. Here we developed protocols for neural fate restriction of mouse PSCs, using knowledge of embryonic development and recent progress with human equivalents. These methodologies rely upon naïve ground-state PSCs temporarily transitioning through LIF-responsive stage prior to neural induction and rapid exposure to regional morphogens. Neural subtypes generated included those of the dorsal forebrain, ventral forebrain, ventral midbrain and hindbrain. This rapid specification, without feeder layers or embryoid-body formation, resulted in high proportions of correctly specified progenitors and neurons with robust reproducibility. These generated neural progenitors/neurons will provide a valuable resource to further understand development, as well disorders affecting specific neuronal subpopulations.

## Introduction

Murine pluripotent stem cells (mPSCs) are a powerful research tool to study development, establish *in vitro* disease models, and facilitate advances in transplantation and drug screens, targeted at neural repair. Since their initial isolation more than three decades ago^1^, mouse embryonic stem cells (mESCs) have been widely used for these aforementioned purposes, however limitations associated with variability and heterogeneity of differentiation protocols have hampered progress.

A key limitation inherrant in early protocols is the reliance on co-culture with stromal cell lines to promote neural induction^2,3^. Although co-culture protocols demonstrate moderate neuralization, variable differentiation efficiencies are unavoidable due to batch-to-batch variation in feeder secreted factors including immunogenic proteins, growth factors and extracellular matrix ligands. While feeder-free neural differentiation alternatives have been developed, they rely on the spontaneous differentiation properties of mESCs in 3-dimensional embyoid bodies (EBs) or 2D cultures, which are inherently variable^4–6^. Specifically, spontaneous differentiation results in contamination with non-neuronal derivatives and/or generates highly heterogeneous cultures. Thus, new protocols to derive specific neural populations under defined culture conditions are warranted.

Studies of fetal CNS development have identified key morphogens involved in the formation of specific brain regions, such as the ventralizing factor sonic hedgehog (SHH) and caudalizing proteins fibroblast growth factor 8 (FGF8) and Wnt1^7^. Significant advancements in the differentiation of human PSCs into restricted neural lineages has come with the early administration of these factors concurrently during neuralization^8–11^, resulting in highly homogenous neural populations that accurately reflect not only CNS regions but also lineage subtypes.

A final consideration is the pluripotent state of the cells at the commencement of differentiation. Two states of mESCs have been described– (i) Serum and LIF (S/L)-dependent ESCs which reflect a more unstable pluripotent state of the pre-implantation blastocyst’s inner cell mass (and most widely employed in ESC studies), and (ii) naïve mESCs, also refered to as ‘ground-state’ stem cells, that represent a stable pre-implantation stage^12^. Naïve state cells are obtained by culturing mESCs in a defined medium (2i) that contains MEK and GSK3ß inhibitors to maintain this ground state and block differentiation signals^13^. These naïve mESCs have numerous advantages over S/L-responsive mESC that are cultured in batch-variable serum-based medium, including greater morphological homogeneity, enriched expression of pluripotential transcription factors and reduced levels of lineage -specific transcripts^14–17^. In this regard, naïve mESC are likely to improve the efficiency and reproducibility of differentiation.

For the first time, we successfully induced neural differentiation of naïve mESCs and compared derivatives to S/L-dependent mESC counterparts. Subsequently, we utilized naïve pluripotent stem cells (PSCs), and advances in hPSC differentiation techniques, to establish methods to derive four region-specific (dorsal forebrain, ventral forebrain, ventral midbrain and hindbrain) neural populations. Of these regions, the ventral forebrain was further divided into rostral and caudal subpopulation, inclusive of ganglionic eminence and hypothalamic-like progenitors, respectively. These (5) novel protocols importantly rely on the early patterning and specification of PSCs using targeted morphogens and small signaling molecules delivered within the first days of neural induction. Extensive cytochemical, cell sorting and transcriptional profiling of the cells confirmed regional specification of resultant progenitors and neurons.

## Experimental Procedures

### Maintenance of PSCs

The mouse ESC lines, E14TG2a (ATCC, USA), and our generated reporter lines (Lmx1a-eGFP and Pitx3-eGFP,^18,19^, as well as the fibroblast-derived induced pluripotent stem cells (IPSCs) line, M2RttA/OKSM, were maintained undifferentiated in either: (i) a basic leukemia inhibitory factor (LIF) medium including serum, or (ii) serum-free 2i medium. Basic LIF medium consisted of Knockout DMEM, 15% fetal bovine serum (FBS, Sigma-Aldrich), 1x penicillin/streptomycin (P/S), 1x glutamax, 1x non-essential amino acids (NEAA), 0.11 mM beta-mercaptoethanol and 2000 IU/ml LIF (Millipore). 2i medium consisted of DMEM/F12, 1x N2 supplement, 1x B27–vitamin A, 1x P/S, 1x glutamax, 1x NEAA, 0.11 mM beta-mercaptoethanol, 2000 IU/ml LIF, 1 uM MEK inhibitor PD0325901 (Stemgent) and 3 uM GSK3 inhibitor CHIR9902 (Stemgent). All PSC were maintained on gelatinized 35 mm dishes under feeder-free conditions. Cells were passaged every second day using accutase (STEMCELL Technologies), for ground state PSC, and 0.025% trypsin-EDTA for S/L-dependent PSC. All reagents were purchased from GIBCO unless stated otherwise.

### PSC differentiation

Naïve PSCs or S/L-dependent PSCs (subsequently refered to as S/L PSCs) were seeded on 0.1% (v/v) gelatinized 48 well plates at a density of 5.0 × 10^3^ cells per well and incubated overnight in LIF basic medium prior to differentiation. A combination of serum replacement medium (SRM) and N2 medium, supplemented with 200 nM LDN193189 (Tocris), were used for early patterning stage under gradient conditions, (day0: 100% SRM; day1: 75%SRM:25%N2; day2: 50%SRM:50%N2; day3: 25%SRM:75%N2). SRM consisted of Knockout DMEM, 1x P/S, 1x glutamax, 1x NEAA, 0.11 mM beta-mercaptoethanol and 15% knockout serum. N2 medium components included DMEM/F12, 1x P/S, 1x glutamax, 1x NEAA, 0.11 mM beta-mercaptoethanol, 1x ITS-A supplement and 1x N2 supplement. This SRM/N2 gradient media was subsequently refered to as ‘Patterning media’. For medial ganglionic eminence differentiation, cells were additionally cultured in a ‘ventralizing media’ from day 6–10, consisting of DMEM/F12, 1x P/S, 1x glutamax, 1x NEAA, 0.11 mM beta-mercaptoethanol, 1x ITS-A, 1x N2 supplement and 1x B27+ vitaminA supplement. Finally, N2B27 medium was utilized for the maturation stage. ‘Maturation medium’ consisted of 1:1 mixture of DMEM/F12 and Neurobasal medium, 1x P/S, 1x glutamax, 1x NEAA, 0.11 mM beta-mercaptoethanol, 1x ITS-A, 1x N2 supplement and 1x B27+ vitamin A supplement.

#### Dorsal forebrain differentiation protocol

For the duration of early patterning (day 0–7), culture media was supplemented with LDN193189 (200 nM, Tocris), with FGF2 (20 ng/ml, Peprotech) added to the media from day 2. Note media was changed daily from day 0–7. Cells were switched to ‘maturation medium’ consisting of: N2B27 supplemented with glial cell-derived neurotrophic factor (GDNF, 30 ng/ml, R&D), brain-derived neurotrophic factor (BDNF, 30 ng/ml, R&D), ascorbic acid (AA, 0.2 mM, Sigma-Aldrich) and the γ-secretase/Notch inhibitor DAPT (10 uM, Tocris) from day 7–14, with media replaced every 2 days, Fig. 3A.

#### Ventral mesodiencephalic differentiation protocol

Ventralization from the default dorsal forebrain phenotype was achieved by modulation of the Shh signaling pathway using Shh recombinant protein (C24II, 200 ng/ml) from day 1–7 of PSC patterning^20^. This supplementation of the media was employed firstly to demonstrate broad neural ventralization of the PSC (resulting in a ‘ventral mesodiencephalic’ population), and was compared to dorsal forbrain and hindbrain regions. Refinement of this ‘ventral mesodiencephalic’ protocol, through the timely delivery of factors to modulate ventralisation (Shh and Wnt) and caudalization (Wnt and FGF8), resulted in more restricted VF fates including rostral VF populations reflective of the ganglionic eminences, a more caudal VF populations inclusive of ‘hypothalamic -like’ neurons and finally a ventral midbrain population. These refined VF and VM protocols are detailed below.

#### Caudal ventral forebrain differentiation protocol

For caudal ventral forebrain (cVF) specification, inclusive of hypothalamic-like neural progenitors/neurons, the patterning medium was supplemented with LDN193189 (200 nM, Tocris), SHH (24CII) (200 ng/ml, R&D) and the smoothened receptor agonist purmorphamine (2 uM, PM) (Stemgent) from day 1–7. From day 3–7, FGF2 (20 ng/ml) was added to the media. On day 7–14, cells were transitioned to maturation medium, Fig. 4A.

#### Rostral Ventral forebrain differentiation protocol

For rostral ventral forebrain (rVF) specification, including ganglionic eminence-like progenitors/neurons, patterning medium was supplemented with LDN193189 (200 nM), and early ventralisation controlled using the Wnt signaling antagonist XAV939 (2 uM, Tocris) from day 0–5. At day 6, medium was supplemented with Shh (200 ng/ml), and PM (2 uM), and changed at day 7 and 9. The medium was switched to maturation medium from day 10–14, with media changes every 2 days, Fig. 5A.

#### Ventral midbrain differentiation protocol

For ventral midbrain specification, SHH (200 ng/ml), PM (2 uM) and FGF8 (25 ng/ml) were added to the patterning media from day 1–7, and further supplemented with CHIR9902 (0.3 uM) from day 2–7, as well as FGF2 (20 ng/ml) on day 3–7. From day 7–14 cells were cultured in maturation medium, Fig. 6A.

#### Hindbrain differentiation protocol

For hindbrain specification, S/L-dependent mouse PSC were cultured in patterning media supplemented with SHH (200 ng/ml) from day 0, with PM (2 uM) and FGF8 (25 ng/ml) added from day 1. On day 2, CHIR99021 (1.5 uM) was added to the media as well as FGF2 (20 ng/ml) at day 3. Media was changed daily from day 0–5. On day 7, differentiated cells were changed into maturation medium, with medium changes every second day until day 14, Fig. 7A.

### Immunocytochemistry and Quantification

Day 0 (undifferentiated PSCs) and day 7, 11 or 14 differentiated cells were fixed and immunostained, using previously described methods^21^. See Supplementary table 1 for primary antibodies. Secondary antibodies, generated in donkey and conjugated to Alexa Flour 488, 555 and 649 were used at 1:200 (Jackson Immunoresearch). 4′,6-diamidino-2-phenylindole (DAPI, SigmaAldrich) nuclear counterstain (1:2000) was used to visualize cells in culture. All the images were captured using a fluorescence microscope (Zeiss Axio Observer Z1).

Quantification of TH+/FoxA2+ and TH+/Pitx3+ (GFP) immunoreactive cells within caudal ventral forebrain and ventral midbrain cultures at day14, established from naïve mESC and iPSC, were performed to differentiate between the dopaminergic neurons generated within these two culture conditions. Using a 20x objective, ten fields of views from three technical, and performed on at least 3 biological replicates were counted. Quantification was performed using Zen Blue software (Zeiss).

### Flow cytometry

Intracellular staining was performed using BD transcription factors staining kit (BD), according to the manufacturer instructions with modifications. In brief, cells (1 × 10^6^/tube) were incubated with BD Horizon fixable viability dye450 (1:1000) diluted in PBS (4 °C, 25 min). Cells were rinsed in PBS, resuspended in fixation buffer (4 °C, 45 min), and washed with ice-cold permeabilization/washing buffer. Fixed cells were incubated with primary antibodies (see supplementary table 1), diluted in permeabilization/washing buffer, overnight at 4 °C. The following day, cells were washed in permeabilization/washing buffer and incubated in secondary antibody (anti mouse-APC,1:500, Santa Cruz), for 45 minutes. After 3x washes in permeabilization buffer, cells were resuspended in ice-cold 500ul running flow buffer consisting of 1% bovine serum albumin (BSA) (Sigma-Aldrich) and 0.5 mM EDTA (Sigma-Aldrich) diluted in PBS. Flow cytometry analysis was performed using Beckman-Coulter CyAn analyzer (Beckman-Coulter), Summit 4.3 software (Beckman-Coulter) and Flowlogic software (Inivai technologies). Single cells were gated based on forward-side scatter profiles and dead cells excluded using violet Horizon viability dye (data not shown). Undifferentiated ESCs were used as a negative control to set gates.

### Quantitative real-time PCR

Total RNA was extracted at day 0, 7 and 14 using Trizol (Ambion). RNA was converted to cDNA and subsequently analyzed using quantitative real-time PCR (qPCR), using previously described methods^22^, to show the expression of 7 pluripotency-related genes (*Nanog*, *Sox2*, *Oct4*, *Rex1*, *Bmp7*, *Wnt7a* and *Id1*) as well as the temporal expression of numerous regionally specified neural-related genes (*Pax6*, *Emx2*, *Nkx2*.*1*, *Gsx2*, *Otp*, *Nhlh2*, *TH*, *En1*, *Nurr1*, *Lmx1a*, *Zic1* and *Hoxa1*). More detailed analysis, to further confirm GE differentiaton, was performed at days 0, 10 and 14, and included the following genes: *Nkx2*.*1*, *Gsx2*, *Lhx2*, *Olig2*, *Dlx2* and *Gad67*. See Supplementary Table 2 for primer sequences.

Student t-tests and One-way *ANOVA*s were performed where appropriate to identify differences within the data, with significance set at p < 0.05.

## Results

### Improved neural differentiation from naïve mESCs

**Figure 1:**
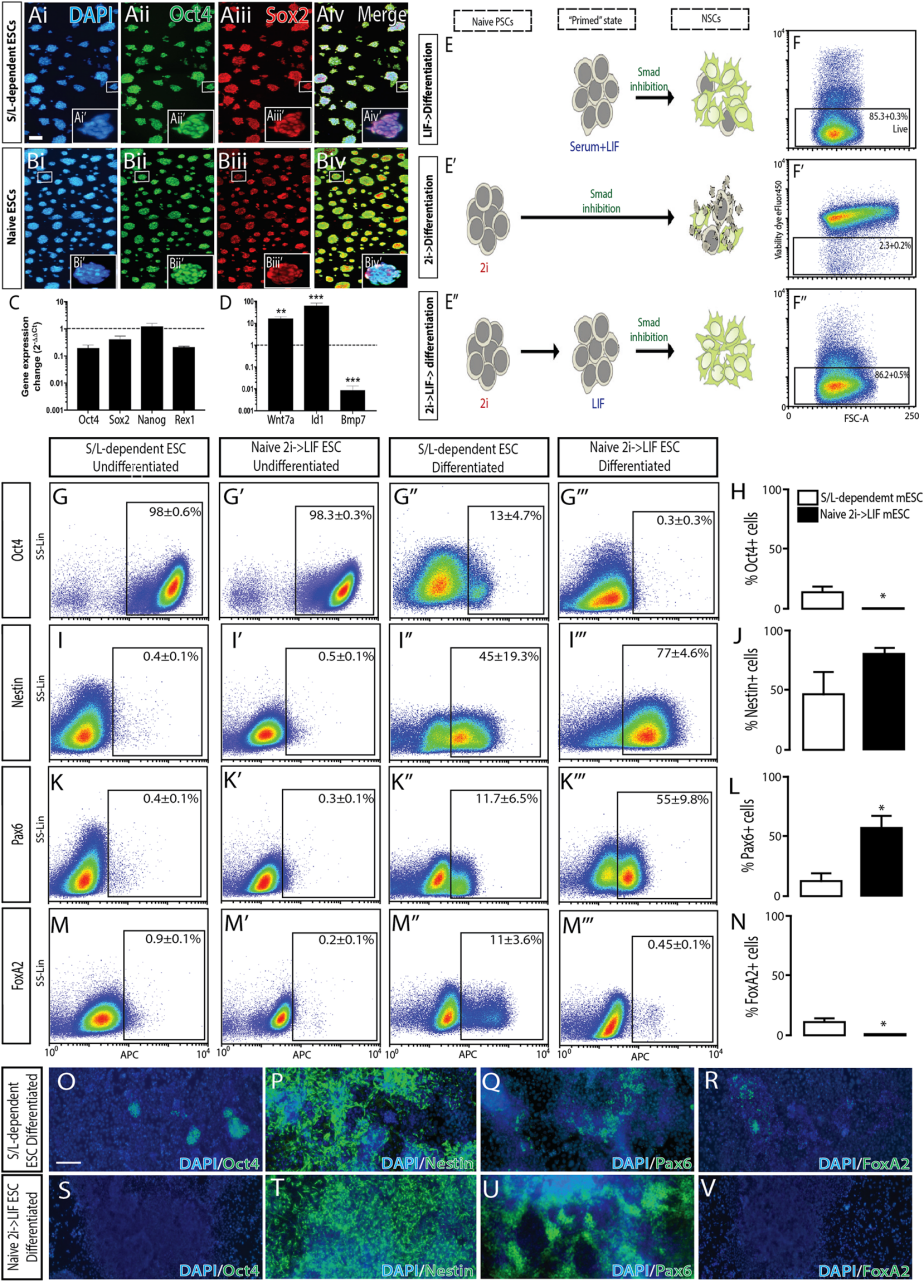
Ground state naïve mESC, compared to serum/LIF-dependent mESC, improve neural specification. S/L-dependent and naïve mESC, labeled with (Ai,Bi) DAPI, show indistinguishable and high expression of pluripotency transcription factors (Aii,Bii) Oct4 and, (Aiii,Biii) Sox2 prior to differentiation. (Aiv,Biv) Merge images. (**C**) Transcriptional profiling confirmed expression of pluripotency genes in undifferentiated naïve and S/L-dependent mESCs. Data represents change in gene expression levels in S/L-dependent mESC (bars), compared to naïve cultures (normalized to 1, dotted line). (**D**) Reflective of differences in pluripotency states, S/L-dependent mESCs showed elevated *Wnt7a* and *Id1*, as well as downregulation of *Bmp7* expression, compared to naïve mESC. (**E**) Schematic depicting the effect of LIF and/or 2i initiated neural differentiation from S/L-dependent verses naïve mESC. (E”) Transient exposure of naïve PSC to LIF resulted in improved neural specification, and elimination of PSC upon differentiation. (**F**) Flow cytometry plots show high viability of differentiated S/L-dependent mESC at day7, (Fi) yet poor survival of naïve mESC differentiation from 2i media, (Eii) an effect that could be circumvented by transient culturing in LIF media. Flow cytometry plots for (**G**) Oct4, (**I**) Nestin, (**K**) Pax6 and (**M**) FoxA2 in undifferentiated and day 7 differentiated cultures. Comparative quantitative analysis of FACS plots for S/L-dependent mESC and naïve mESCs at day7 after differentiation for (H) Oct4, (**J**) Nestin, (**L**) Pax6 and (**N**) FoxA2. Upon differentiation, naïve ESC cultures showed reduced proportions of contaminating Oct4+ PSC, elevated neuralization (Nestin+) and appropriate default dorsal forebrain specification, as indicated by reduced FoxA2+ and elevated Pax6+ cells in culture. Representative images of (**O**,**S**) Oct4, (**P**,**T**) Nestin, (**Q**,**U**) Pax6 and (**R**,**V**) FoxA2 immunostaining from primed and naïve mESCs differentiated under neural conditions for 7days. Note the absence of both Oct4+ PSCs and FoxA2 off-target ventral neural progenitors, as well as expansive Nestin+ and Pax6+ NPC in cultures derived from naïve mESC, compared to S/L-dependent mESC. Data (n = 4) represents mean ± SEM, Students t-test. Scale bars: A-B = 100 um, O-V = 50um. *p < 0.05, **p < 0.01, ***p < 0.001. mESC: mouse embryonic stem cell; NPC: Neural pluripotent stem cells; PSC: pluripotent stem cells; S/L: Serum and LIF.

S/L-dependent mESCs (cultured in serum and LIF medium^1^) and naïve mESCs (cultured in 2i medium^13^), were first examined by immunocytochemistry to confirm robust expression of cardinal pluripotent transcription factors, Oct4 and Sox2 (Fig. 1A,B). Consistent with this observation, quantitative PCR revealed similar high levels of expression for the pluripotency genes *Oct4*, *Sox2*, *Nanog* and *Rex1* in both S/L-dependent and naïve mESC cultures (Fig. 1C). Further, flow cytometry quantification could not statistically distinguish the two mESC populations, with ≥98% of cells immunoreactive for Oct4 (Fig. 1G,G’). While naïve ground state and S/L-dependent PSC displayed similarities in their expression of pluripotent genes, recent studies have recognized transcriptional differences between these two pluripotent staged cell populations^15^. In concerrence, we show significantly elevated expression of *Wnt7a* and *Id1*, as well as a down regulation of Bmp7, in our S/L-dependent mESCs compared to naïve mESCs, Fig. 1D.

S/L-dependent and naïve mESCs were directed towards a neural fate by antagonism of Smad signaling, similar to that widely employed for monolayer-based differentiation cultures of human PSC, Figure 1E,E’^23^. This minimalist default differentiation system produced a heterogeneous population of cell types from S/L-dependent mESCs following 7 days of differentiation, including Nestin immunoreactive cells indicative of NPCs (45 ± 19.3%, Fig. 1I”,J,P), some of which possessed a dorsal forebrain identity as shown by expression of the dorsal marker Pax6 (11.7 ± 6.5%, Fig. 1K”,L,Q), or a ventral identity determined by expression of the floor-plate marker FoxA2 (11 ± 3.6%, Fig. 1M”,N,R).

Differentiation of naïve mESCs under the same conditions was significantly compromised by excessive cell death and limited NPC fate acquisition (Fig. 1E’,F’). We speculated these complications arose from directing naïve pluripotent cells into a neural fate immediately. To circumvent this, 2i cultured naïve mESCs were subjected to a 24-hour incubation in LIF-primed media prior to Smad inhibitor exposure (Fig. 1E”) to mimic the graduated transition that occurs during embryonic development from the pre-implantation (naïve pluripotency) to post-implantation stage, and then to germ layer restriction.

Indeed, differentiated naïve mESCs that incorporated a LIF-primed transition showed high viability (>86%, Fig. 1F”), efficiently neuralized (77 ± 4.6% Nestin expression, Fig. 1I”’,J) and generated a homogenous population of NPCs (Fig. 1T). Although the increase in Nestin expression in naïve-LIF-differentiated cultures was not significant compared to differentiation of S/L-dependent cultures (Fig. 1J), patterning from a naïve state was drastically less variable (Fig. 1P,T), and contained extremely few OCT4-positive cells (0.3 ± 0.3% vs 13 ± 4.7% Oct4+, Fig. 1G”,G”’,H,O,S). Furthermore, naïve-LIF-differentiated cultures acquired a more reproducible and restricted phenotype, with Pax6 expressed in 55 ± 9.8% of day 7 cultures (Fig. 1K”’,L,U), and the absence of off-target floor-plate progenitors marked by FoxA2 (Fig. 1M’”,N,V). These results demonstrate for the first time a feeder-free monolayer directed differentiation platform for the robust production of NPCs from naïve mESCs.

It is important to note that neuralization via Smad inhibition was achieved by blocking only one of two major Smad families. Specifically, the BMP inhibitor LDN193189 was used to block Smads 1/5/8 while the TGF-β pathway, that regulates Smad 2/3 signaling and is typically co-inhibited in human neural differentiations, was not modulated. This was due to our observation that Smad 2/3 inhibition (using SB431542) prevented naïve mESC exiting from their pluripotent state, with cells showing maintained Oct4 expression (data not shown) – a finding that supports previous studies describing the necessity for Smad 2/3 suppression in the maintenance of ground state identity^24^.

### Bi-directional regional neural specification

**Figure 2:**
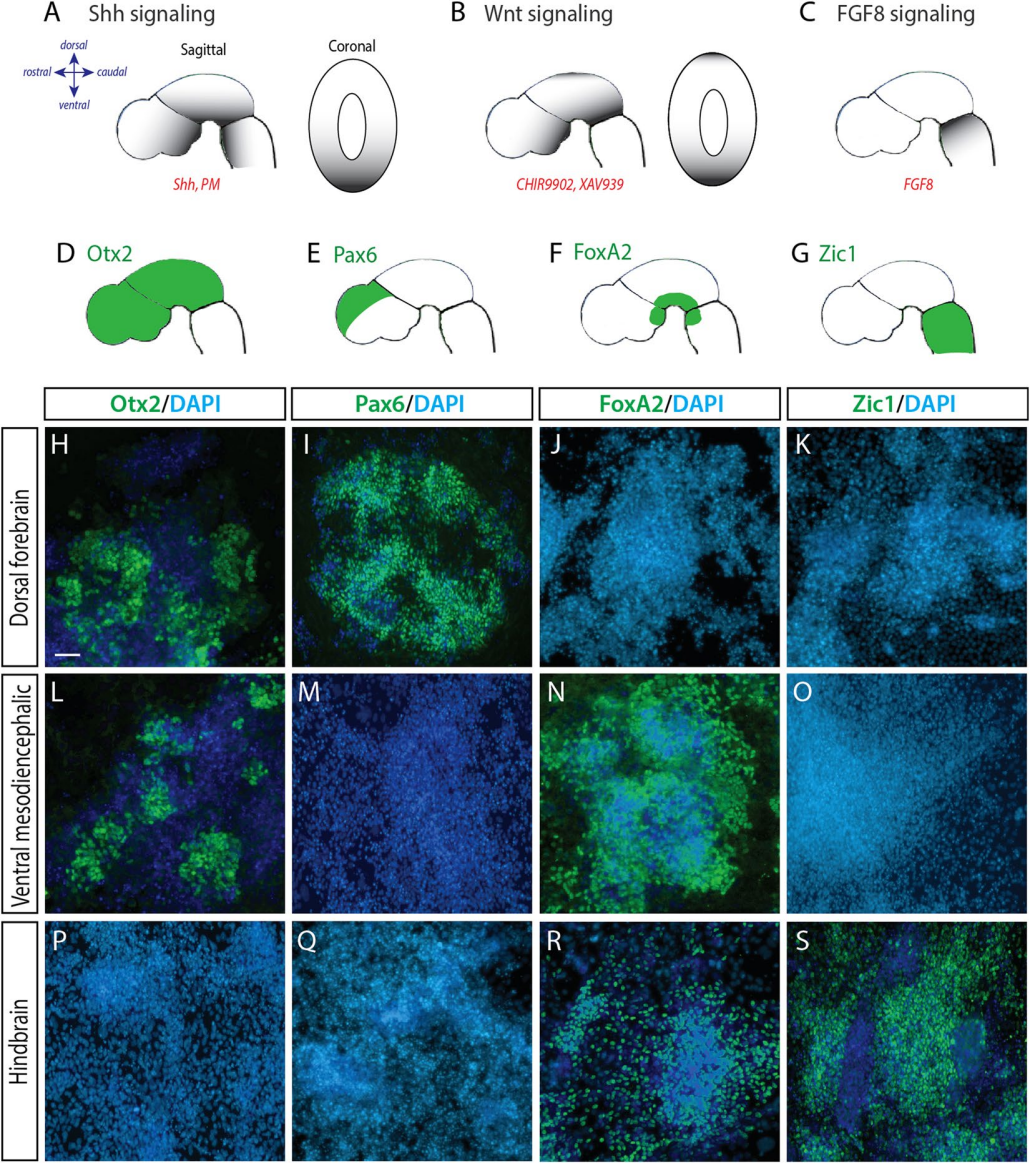
Modulation of bi-directional morphogen gradients generates regionally specified neural progenitors. Developing embryo schematics illustrating morphogen gradients (grey shading) and the utilized protein/small moleulces (red) employed to modulate (**A**) Shh, (**B**) Wnts and (**C**) FGF8. Schematic illustrations of the embryo (sagittal plane) depicting expression of key transcription factors (**D**) Otx2, (**E**) Pax6, (**F**) FoxA2 and (**G**) Zic1, used to identify rostral forebrain-midbrain, dorsal forebrain, ventral, and caudal hindbrain progenitors, respectively. (**H**–**K**) Representative photomicrographs of naïve mESC differentiated under default neural conditions to generate dorsal forebrain progenitors, expressing Otx2 and Pax6, but lacking FoxA2 and Zic1 expression. (**L–O**) Naïve mESC differentiated towards ventralised mesodiencephalic and (**P**–**S**) hindbrain progenitor fates, showing appropriate Otx2, Pax6, FoxA2 and Zic1 expression indicative of regional specification. Scale bar = 50 um.

In light of the improved reproducibility and homogenicity of neural induction from naïve, compared to S/L-dependent, mESC cutlures we next assessed the capacity for ground state cells to be redirected along developmental dorso-ventral and rostro-caudal axes using a range of small molecules and morphogens to mimic *in vivo* embryonic signals and obtain dorsal forebrain, ventral mesodiencephalic and hindbrain cultures (Fig. 2). Naïve mESCs differentiated in basal Smad inhibition conditions (LDN193189 from day 0–7) resulted in the ‘default’ acquisition a dorsal forebrain identity with progenitors expressing the forebrain-midbrain marker Otx2, dorsal transcription factor Pax6, as well as absence of the ventral marker, FoxA2, and hindbrain marker, Zic1 (Fig. 2D–K). Ventralization from this dorsal phenotype was achieved with the addition of Shh (day 1–7), causing a predicted loss of Pax6 expression, upregulation of FoxA2, and maintenance of Otx2 identity (Fig. 2L–O). In the absence of caudalising cues, the resultant population was a heterogeneous pool of both ventral diencephalic and ventral mesencephalic progenitors, here termed ‘ventral mesodiencephalic’, Fig. 2L–O.

**Figure 3:**
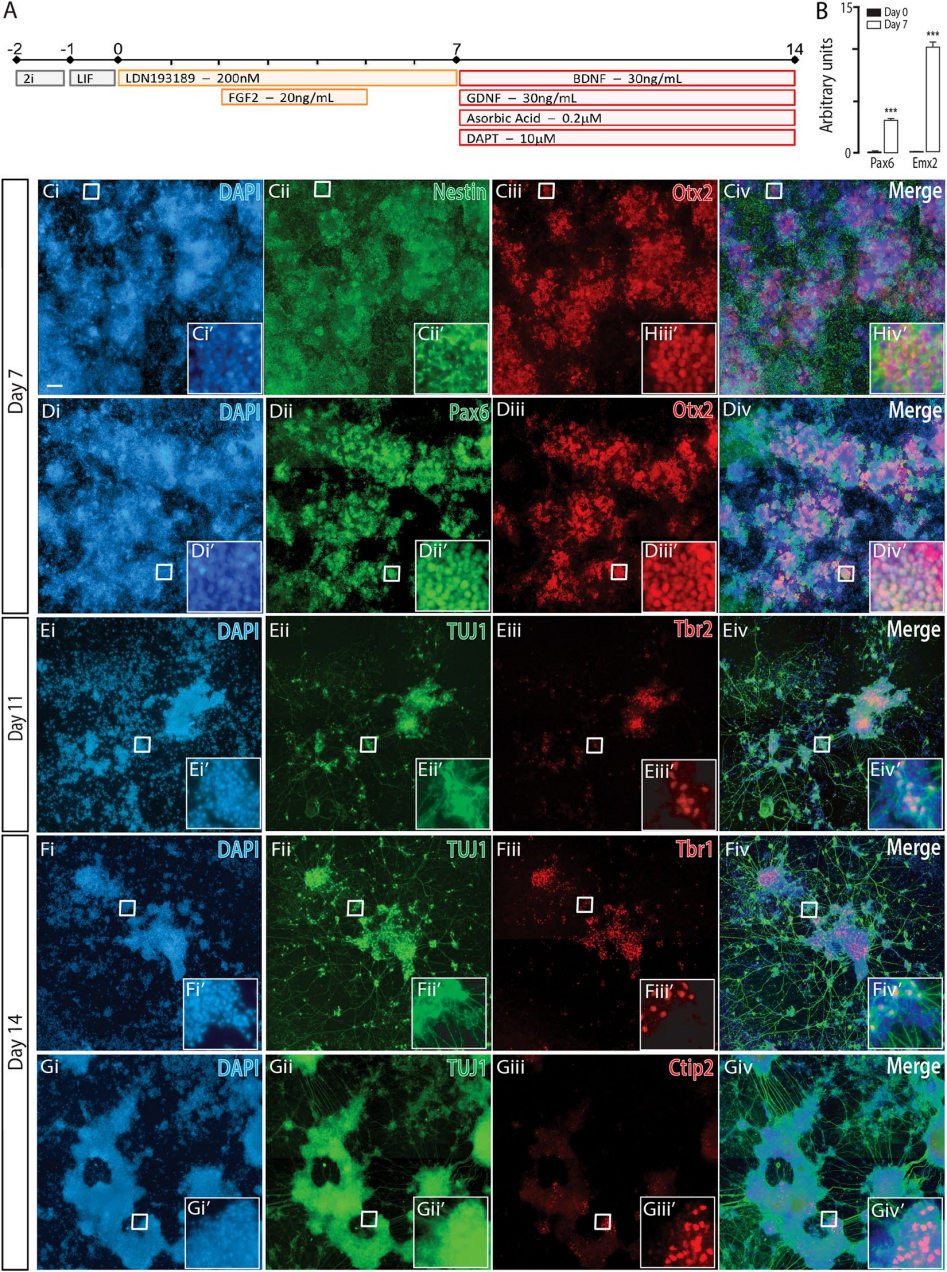
Naïve mESC, differentiated to a dorsal forebrain fate, show appropriate regional and temporal specification (**A**) Neural differentiation protocol detailing morphogens and small molecules employed to generate dorsal forebrain progenitors and neurons from naïve PSCs. (**B**) Quantification of transcriptional expression of dorsal forebrain indicative genes *Pax6* and *Emx2* in undifferentiated (day 0, black bar) and day 7 differentiated cultures (white bar). (**C**,**D**) Representative micrographs depicting expression of Nestin, Otx2 and Pax6 in dorsal forebrain progenitors at day7. (**E**) Maturing dorsal forebrain neurons, expressing Tuj1, show transient Tbr2 expression at day 11, and mature dorsal forebrain markers inclusive of (**F**) Tbr1 and (**G**) Ctip2 by day 14. Images show culture overviews, while inserts show immunocytochemical labeling at the resolution of individual cells. Data (n = 4) represented as mean ± SEM, Students t-test. Scale bar = 100 um. ***p < 0.001.

**Figure 4:**
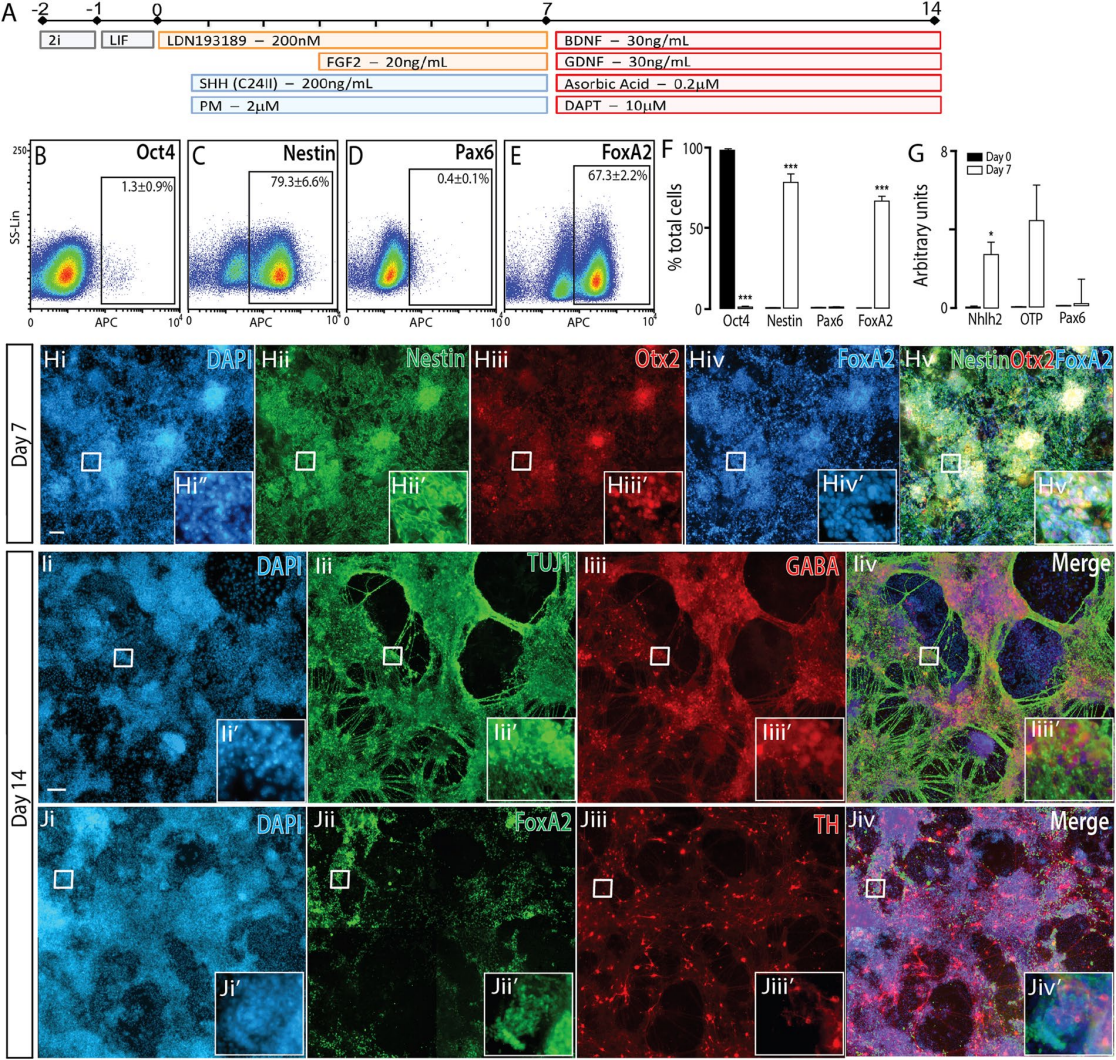
Naïve mESCs differentiated to a caudal ventral forebrain fate, show appropriate regional and temporal specification. (**A**) Neural differentiation protocol, detailing morphogen and small molecules employed, to generate ventral forebrain progenitors and neurons from naïve PSC. (**B**–**E**) Flow cytometry plots at day 7 and, (**F**) quantitative analysis at 0 and 7 for Oct4, Nestin, Pax6 and FoxA2, showing appropriate downregulaton of pluripotent (Oct4) and dorsal (Pax6) markers together with upregulation of neural (Nestin) and ventral (FoxA2) protein expression. (**G**) Transcriptional downregulation of *Pax6* together with the upregulation of *Nhlh2* and *OTP* suggested these cultures included neural progenitors of a ventral hypothalamic fate. (**H**) Representative photomicrographs depicting expression of Nestin, Otx2 and FoxA2 in ventral forebrain progenitors at day7. (**I**) Maturation of cultures resulted in the generation of GABA+ TUJ+ neurons, as well as FoxA2+ and TH+ neurons (showing low co-localisation), suggestive of hypothalamic neurons. Data (n = 3) represents mean ± SEM, Students t-test. Scale bar = 100 um. *p < 0.05, ***p < 0.001.

**Figure 5:**
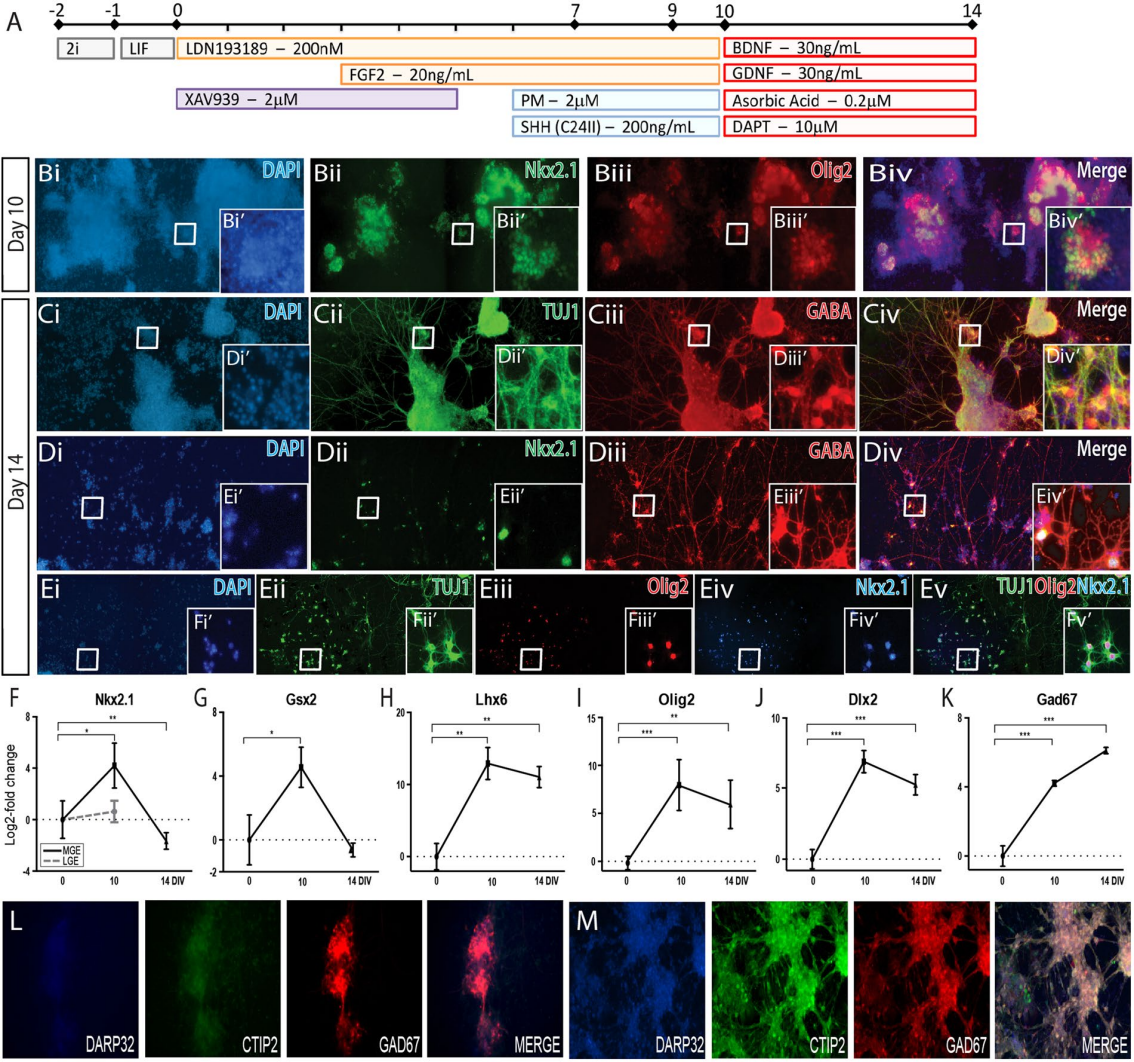
Naïve mESCs differentiate to rostral ventral forebrain/GE progenitors and mature GABAergic neurons. (**A**) Schematic of the neural differentiation protocol, detailing morphogen and small molecules employed, to generate medial ganglionic eminence progenitors and neurons from naïve PSCs. (**B**) Immunocytochemistry images highlighting the expression of key GE transcription factors Nkx2.1 and Olig2 within young post-mitotic cell at day 10 of differentiation. (**C**) mESCs differentiated to GE-specific neurons widely expressed TUJ1 and GABA, (**D**) with many GABA + neurons co-expressing Nkx2.1, indicative of MGE interneurons. (**E**) Numerous post-mitotic TUJ1+ neurons co-expressed the MGE markers Nkx2.1 and Olig2 by day 14. Note images (**B**–**E**) show cultures overviews, illustrating the largely homogeneous nature of the differentiations, while inserts (Bi’-Ev’) show immunocytochemical labeling at the resolution of individual cells. Numerous GE-related genes showed appropriate temporal expression, including the transient upregulation of (**F**) *Nkx2*.*1* and (**G**) *Gsx2* as well as maintained, elevated expression of **(H**) *Lhx6*, (**I**) *Olig2* and (**J**) *Dlx2*. (**K**) *Gad67* was significantly upregulated by GE-like progenitors at day 10, with expression peaking at day 14 in mature neurons. Dashed grey line in (**F**) represents Nkx2.1 expression in “LGE-like” cultures, induced by earlier ventralization. (**L**) Reflective of an ‘MGE-like’ fate, rostral VF cultures appropriately expressed GAD67, but not CTIP2 or DARPP32. (**L**) In contrast, earlier ventralization of these VF progenitors resulted in cultures showing high expression of DARPP32, CTIP2 and GAD67, suggestive of a possible ‘LGE-like fate. Scale bar = 100 um. Data (n = 3) represented as mean ± SD. One-way *ANOVA* with Tukey post-hoc test. *p < 0.05, **p < 0.01, ***p < 0.001. MGE: medial ganglionic eminience; LGE: laternal ganglionic eminence.

**Figure 6:**
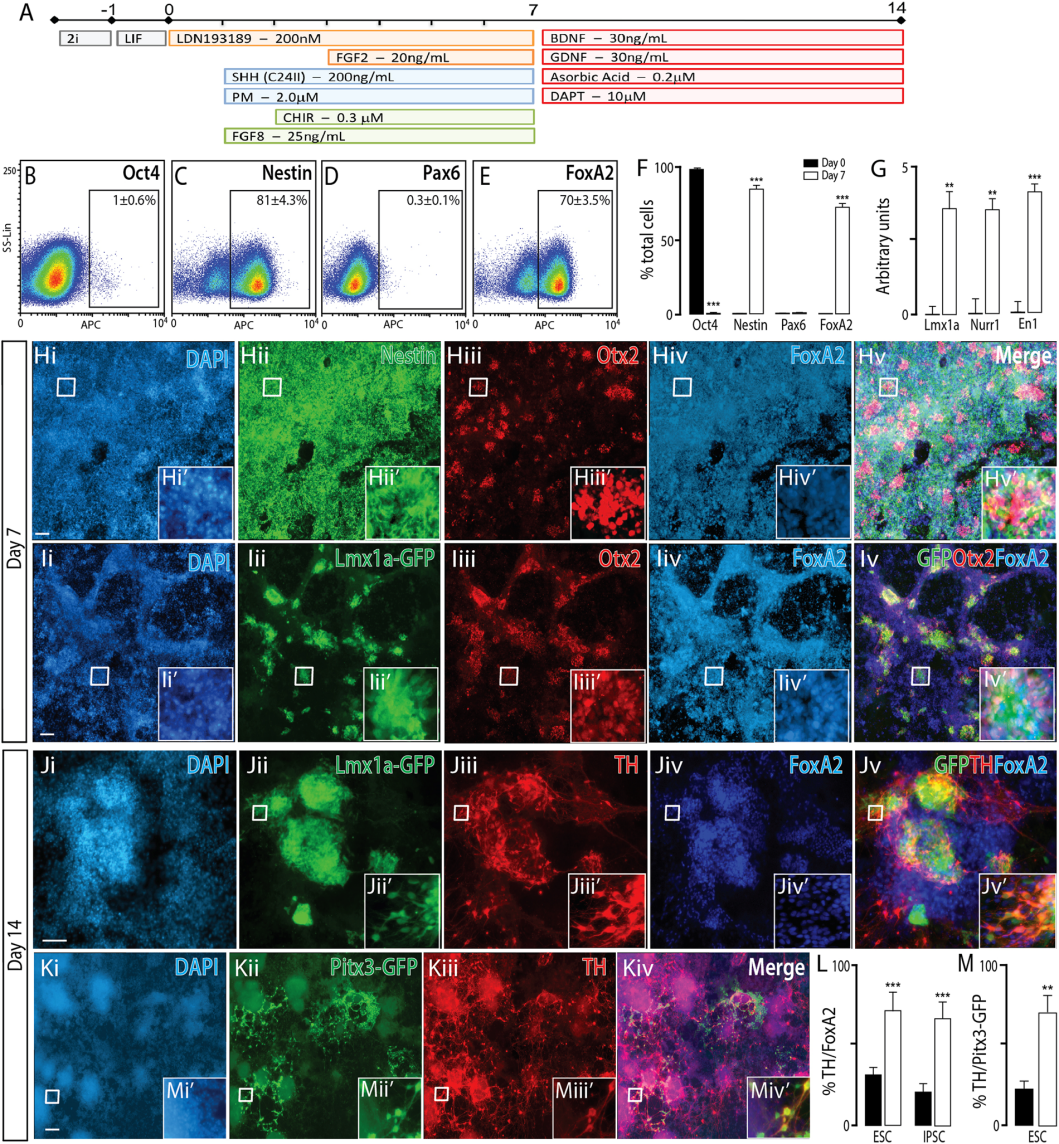
Naïve mESC differentiated to a ventral midbrain fate, show appropriate regional and temporal specification. (**A**) Schematic of the neural differentiation protocol, detailing morphogen and small molecules employed, to generate ventral midbrain progenitors and neurons from naïve PSC. (**B**–**E**) Flow cytometry plots at day 7 and, (**F**) quantitative analysis at day 0 and 7 for Oct4, Nestin, Pax6 and FoxA2, showing appropriate downregulaton of pluripotent (Oct4) and dorsal (Pax6) markers together with upregulation of neural (Nestin) and ventral (FoxA2) expression. (**G**) Transcriptional expression of ventral midbrain genes *Lmx1a*, *Nurr1* and *En1*. (**H**) Representative photomicrographs depicting expression of Nestin, Otx2 and FoxA2 in VM progenitors at day7. (**I**) Employment of an Lmx1a-GFP reporter line demonstrated high co-expression with Otx2 and FoxA2, indicative of ventral mesencephalic progenitors. (**J**) By day 14, these progenitors matured into VM dopaminergic neurons co-expressing the hallmark proteins Lmx1a, TH and FoxA2. (**K**) Representative images depicting Pitx3-GFP and TH expression within mature cultures, confirming ventral midbrain dopaminergic fate. (**L**) Quantification of %TH/FoxA2 neurons from VM (white) and ventral forebrain (black bars) cultures. Elevated proportions of TH+ FoxA2+ co-expressing neurons confirmed a VM dopaminergic phenotype, while FoxA2+ TH+ cells within VF cultures were indicative of off-target populations. (**M**) Quantification of TH+ /Pitx3-GFP+ neurons within ventral forebrain and ventral midbrain cultures at day 14, using the Pitx3-GFP reporter mESC line, confirmed the generation of *bona fide* VM dopaminergic neurons within VM cultures. Data represents mean ± SEM, n = 3, Students t-test. Scale bar = 100 um. **p < 0.01, ***p < 0.001.

**Figure 7:**
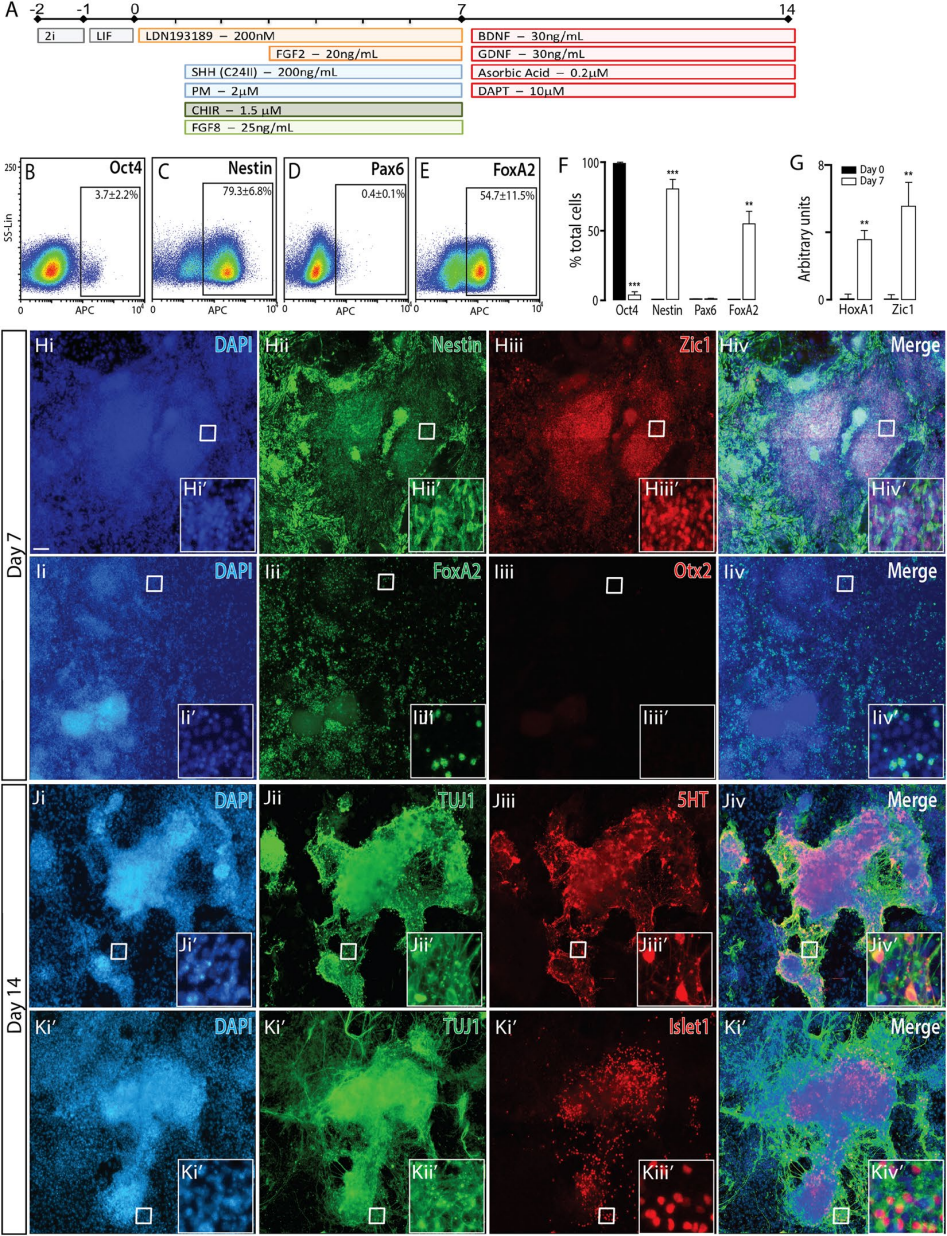
Naïve mESCs differentiated to a ventral hindbrain fate, show appropriate regional and temporal specification. (**A**) Schematic of the neural differentiation protocol, illustrating morphogen and small molecules employed, to generate ventral hindbrain progenitors and neurons from naïve PSCs. (**B–E**) Flow cytometry plots at day 7 and, (**F**) quantitative analysis at 0 and 7 for Oct4, Nestin, FoxA2 and Pax6, showing appropriate downregulaton of pluripotent (Oct4) and dorsal (Pax6) markers together with upregulation of neural (Nestin) and ventral (FoxA2) expression. (**G**) Transcriptional expression of hindbrain genes *Hoxa1* and *Zic1*. (**H**) Representative photomicrographs depicting expression of DAPI, Nestin and Zic1 in hindbrain progenitors at day 7. (**I**) Ventral hindbrain specification of progenitors was confirmed by the presence of ventral marker FoxA2 and absence of mesodiencephalic protein Otx2. (**J**) By day 14, hindbrain specified cultures contained serotinergic neurons immunoreactive for TUJ1 and 5HT, as well as (**K**) Islet1+ TUJ1+ neurons, indicative of motor neurons. Data represents mean ± SEM, (n = 3), Students t-test. Scale bar = 100 um. **p < 0.01, ***p < 0.001.

**Figure 8:**
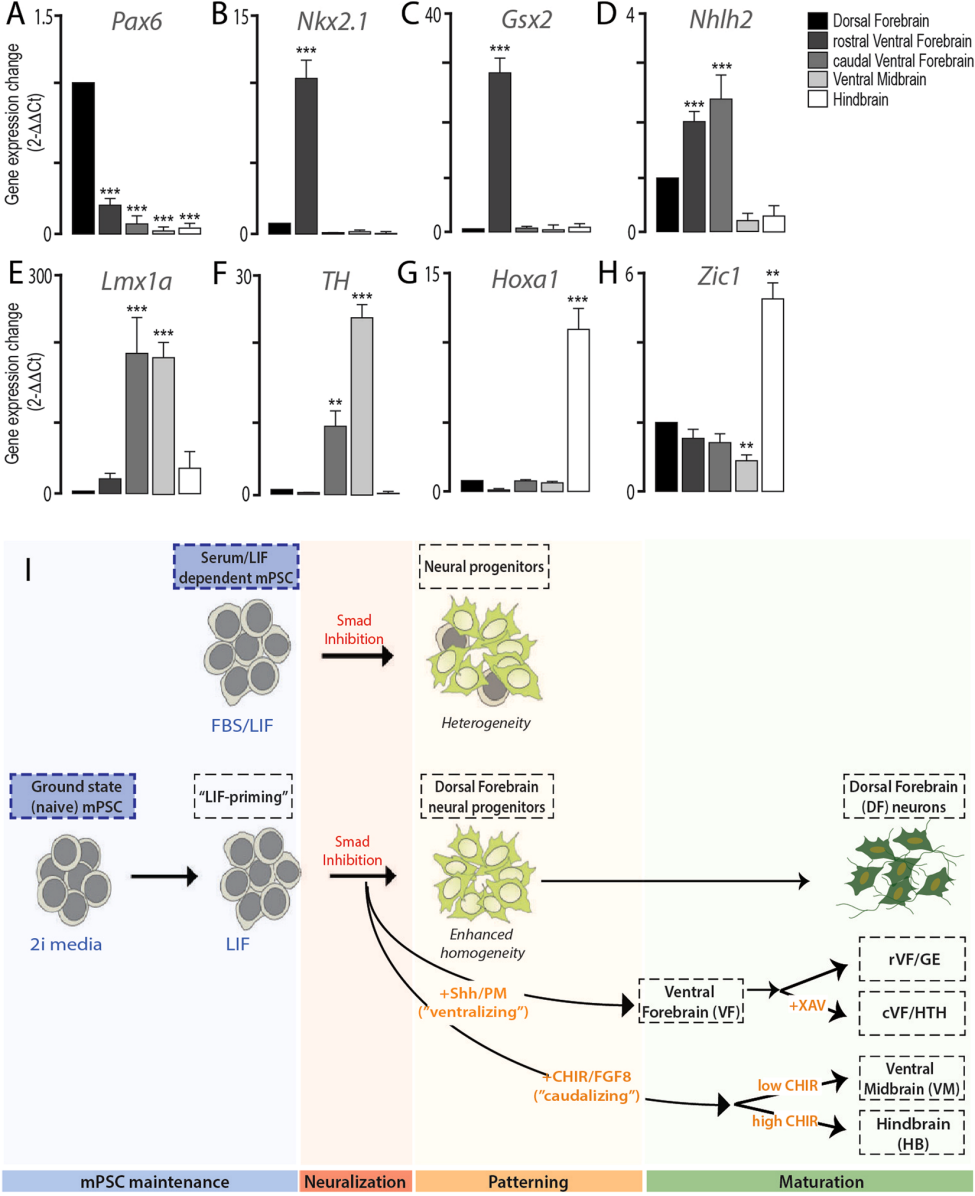
Transcriptional profiling of regionally distinct neural progenitor populations from mESC, and overview of differentiation. Transcriptional profiling of five neural progenitor populations, derived from naïve, ground state mESC, assessing key patterning and regionally localised genes including (**A**) *Pax6*, (**B**) *Nkx2*.*1*, (**C**) *Gsx2*, (**D**) *Nhlh2*, (**E**) *Lmx1a*, (**F**) *TH*, (**G**) *Hoxa1* and (**H**) *Zic1*. Upregulation of select genes within given populations confirmed specificity of the differentiations. (**I**) Schematic overview illustrating the “priming” of ground state pluripotent stem cells (blue shading), to promote homogeneneous neuralisation (pink) of mouse PSC. Subsequent patterning (orange) and maturation of cells (green), through the time and dose-dependent administration of morphogens and small molecules, resulted in the generation of regionally distinct neuronal populations including those of the dorsal forebrain (DF)/cortex, rostral and caudal ventral forebrain (rVF, cVF), ventral midbrain (VM) and hindbrain (HB).

The addition of high concentrations of the GSK3ß inhibitor and potent caudalizing small molecule CHIR9902 (day 2–7), as well as FGF8, was sufficient to push culture identity beyond the isthmic organizer, resulting in the generation of hindbrain NPCs, as determined by upregulation of Zic1 and surpression of Otx2 expression (Fig. 2P–S). Each of these regionally patterned populations (dorsal, ventral and caudalized) were further modulated, matured and characterized in subsequent Figs (3–8).

### Naïve PSC differentiated to dorsal forebrain neurons

Confirming the dorsal forebrain progenitor identity seen by immunocytochemistry (Otx2+, Pax6+, Nestin+, Figs 2H–I and 3C,D), we observed elevated transcript levels of *Pax6* and the cortical lineage marker *Emx2* ^25^, by day 7 (Fig. 3B). These progenitors were driven to a post-mitotic fate by the removal of NPC morphogens and growth factors (LDN193189 and FGF2), in conjunction with the addition of the Notch pathway small molecule inhibitor DAPT^26^, and pro-neuronal survival trophins including BDNF, GDNF and Ascorbic Acid (Fig. 3A). Following 4 days of maturation (day 11), the emergence of the neuronal cytoskeletal protein TUJ1, with the intermediate cortical progenitor marker Tbr2, were seen (Fig. 3E). These results mirror cortical developmental where transient Tbr2 expression follows Pax6 expression before radial glial cells commit to specific laminar layers^27^. With extended culturing in maturation conditions, increased numbers of Tuj1+ post-mitotic neurons were observed and co-expressed the mature cortical laminar layer markers Tbr1 (Fig. 3F) and Ctip2 (Fig. 3G). Importantly, we were able to demonstrate the ability to robustly reproduce these findings, also differentiating naïve ground state iPSCs to a dorsal forebrain fate (Supplementary Figure 1).

### Naïve PSC differentiated to ventral forebrain neurons

While modulation of the Shh signaling was shown to impact default dorsal forebrain identity of PSC, resulting in acquisition of ventral neural tube fate (Fig. 2), minimal assessment of the directed differentiation and resultant progenitors/neurons was performed. Here we importantly demonstrate that ventralized cultures maintained the robust downregulation of Oct4 and upregulation of the NPC identity marker Nestin (Fig. 4B,C,F). As anticipated, ventral signals induced the loss of dorsal (Pax6) identity (Fig. 4D,F,G) and significant upregulation of the ventral neural tube marker FoxA2 (67.3 ± 2.2%, Fig. 4E,F). The ventral forebrain is the site of hypothalamus formation, and indeed hypothalamic transcripts *Nhlh2* and *Otp* were significantly up-regulated under these conditions^28,29^, suggesting the adoption of a diencephalic fate (Fig. 4G). By maturating ventral forebrain NPCs, TUJ1 post-mitotic neurons arose throughout cultures with significant GABA expression (Fig. 4I), further supporting hypothalamic-like identity. While numerous FoxA2 and TH + cells were observed by day 14, these populations showed minimal overlap, reflective of VF hypothalamic dopaminergic neurons (as opposed to FoxA2 + /TH + dopamine neurons that reside in the adjacent VM) (Fig. 4J and Supplementary Figure 2),^30^.

Interestingly, these ventral forebrain cultures did not express transcription factors Nkx2.1 or Olig2 (data not shown), *in vivo* markers of the adjacent developing ganglionic eminence, GE. Early developmental studies have demonstrated that inhibition of Wnt/beta-catenin signaling is important for the up-regulation of key GE transcription factors such as *Dlx2*, *Mash1* and *Gsx2*, and suppression of pallial markers such as *Emx2* ^31^. Thus, we theorized the addition of a Wnt inhibiting small molecule (XAV939) may induce the generation of GE identity. Indeed, in response to XAV, efficient Nestin+ and Otx2+ forebrain NPCs were observed (Supplementary Figure 3) in conjunction with broad expression of Nkx2.1 and Olig2 (Fig. 5B, Supplementary Figure 4), suggestive of a GE phenotype.

Continued maturation promoted widespread co-expression of TUJ1 and GABA (Fig. 5C,D and Supplementary Figure 4) that may represent lateral ganglionic eminence (LGE)-derived striatal neurons or MGE-derived interneurons. This former population can be identified by the co-expression of DARPP32+/CTIP2+ (that were not present within these cultures, Fig. 5L), and the latter, MGE neurons, confirmed by co-expression of GABA+/NKX2.1+ and NKX2.1+/OLIG2+ (Fig. 5D–E, Supplementary Figure 4). Validating these findings, transcript assessment showed significant upregulationof a raft of MGE-related NPC genes including *Nkx2*.*1*, *Gsx2*, *Lhx6*, *Olig2* and *Dlx2* (Fig. 5F–J), (Corbin *et al*., 2000) and upregulation with maturation of Gad67, an enzyme important in GABA synthesis for GE neurons (Fig. 5K). Of interest, in the development of this rVF protocol we have observed that earlier ventralisation of the cultures (though the administration of Shh+ PM from day 3–7) resulted in cells co-expressing DARPP32+ CTIP2+ GAD67+ (Fig. 5M) and comparatively low Nkx2.1 transcript compared to the “GE/MGE-like” populations (Fig. 5F, grey line), suggestive of a possible “LGE-like” fate (Fig. 5M). Taken together, this data indicates GE-like NPCs and neurons from ground state mPSC.

### Naïve PSC differentiated to ventral midbrain neurons

During embryonic development, the isthmic organizer, at the midbrain-hindbrain boundary, secretes FGF8 and Wnts to instruct the formation of the mesencephalon^32^, Fig. 2B,C, including important populations of dopaminergic neurons. To generate ventral midbrain (VM) NPCs, our caudal ventral forebrain protocol was supplemented with FGF8 (25 ng/ml) and the Wnt agonist CHIR9902 (0.3 uM). This was seen to maintain cultures rich in NPC (81 ± 4.3% Nestin+, Fig. 6C), that expressed appropriately high levels of regional identity proteins FoxA2 (70 ± 3.5%, Fig. 6E,F,H) and the forebrain-midbrain marker Otx2 (Fig. 6H,I). Further, these cultures upregulated essential VM dopaminergic transcripts *Lmx1a*, *Nurr1* and *En1* (Fig 6G)^32^. To further confirm aVM identity of these NPCs we utilized a mESC reporter line for Lmx1a (Lmx1a-eGFP); a transcription factor expressed in the roof plate and throughout the developing floor plate. In combination with Otx2 and FoxA2, the mesendiencephalic floor plate can be distinguished from roofplate NPCs and ventral hindbrain, and was seen in differentiating cultures derived from naïve mESCs (Fig. 6I).

Ongoing maturation of the cultures to day 14, resulted in the presence of many tyrosine hydroxylase (TH – the rate-limiting enzyme in dopamine synthesis) expressing neurons (Fig. 6J,K). To differentiate between dopaminergic neurons of the caudal ventral forebrain and the ventral midbrain, we quantified TH+/FoxA2+ neurons (a population restricted to VM dopaminergic neurons). Under VM differentiation conditions, the majority (>75%) of TH+ neurons co-expressed FoxA2 (Fig. 6J,L and Supplementary Figure 5). VM dopamine neurons were characterized further using both the Lmx1a-eGFP and Pitx3-eGFP knockin reporter line that mark *bona fide* VM DAs^33^. In terminally differentiated cultures neurons co-expressing TH, Lmx1a and FoxA2 were observed (Fig. 6J), with the majority of TH+ neurons (69 ± 10.4%) co-expressing Pitx3-GFP (Fig. 6K,M). By comparison, ventral forebrain differentiation of the Pitx3-eGFP mESC reporter line saw significantly less TH+/FoxA2+ (28 + 4%, Fig. 6L), and Pitx3-GFP+/TH+ co-expressing neurons (22 ± 6%, Fig. 6N), both likely off-target populations within the VF differentiations, yet overall importantly highlight the capacity to direct naïve mPSC into ventral diencephalic or mesencephalic dopaminergic populations.

### Naïve PSC differentiated to ventral hindbrain neurons

Upon successful generation of VM progenitors and neurons, we finally questioned whether stronger activation of the canonical Wnt pathway would be sufficient to driving NPCs beyond the isthmus and adopt a more caudal, hindbrain phenotype. Indeed, 5-fold higher CHIR9902 concentrations (1.5 uM), in conjunction with Shh signaling, resulted in high yields of neural progenitors (79.3 + 6.8% Nestin+), that lacked rostral (Otx2, Fig. 7Iiii) and dorsal (Pax6, Fig. 7D,F) identity markers, and showed appropriate ventralization (54.7 + 11.5% FoxA2+, Fig. 7E,F,I) at day 7. More specifically, day 7 cultures showed upregulated transcript levels of two key hindbrain genes, *Hoxa1*, crucial for the early patterning of the rhombencephalon^34^ and *Zic1*, a key regulator of progenitor proliferation during early cerebellar development^35^, Fig. 7G,H. Following maturation, immunocytochemical analysis at day 14 revealed the presence of numerous 5-HT+ serotonergic neurons, a population enriched throughout the hindbrain^36^, (Fig. 7J, Supplementary Figure 6) as well as hindbrain Islet1+ motor neurons (Fig. 7K, Supplementary Figure 6),^37^. Taken together, these observation strongtly indiciate ventral hindbrain NPC generation from naïve PSC under these ventro-caudalizing conditions.

In support of the capacity for these 5 described protocols to generate neural progenitors and neurons with relatively restricted regional identity, we compared transcript levels for genes known to be expressed within these areas in embryonic development, and their absence (or appropriately comparative levels) from other fate specified populations. Given the ‘default’ generation of dorsal forebrain progenitors/neurons upon Smad-induced neuralisation of PSC, gene expression within ventral and caudalised populations was compared to this DF population. As such, we demonstrated the dorsal forebrain gene, *Pax6*, was significantly downregulated in all 4 other neural populations (Fig. 8A). Ganglinic eminence genes, *Nkx2*.*1* and *Gsx2*, were strongly expressed only in rostral ventral forebrain cultures (Fig. 8B,C). Within caudal ventral forebrain, inclusive of hypothalamic progenitors, *Nhlh2* was notably increased, and significantly elevated compared to more caudal VM and HB populations (Fig. 8D). *Nhlh2* however was also shown to be upregulated within the adjacent rostral VF (GE-like) cells, expression that may be explained by its known identity within subependymal neural progenitors. Reflective of *in vivo* neural development and the localization of dopaminergic subpopulations, *Lmx1a* and *TH* gene expression were elevated in caudal ventral forebrain (cVF) and VM progenitors and lowly expressed in more rostral (DF, cVF) and caudal (hindbrain) cultures (Fig. 8E,F). Finally, the rhombencephalic related genes *Hoxa1* and *Zic1* were only upregulated in HB cultures (Fig. 8G,H).

### Significance Statement

Mouse pluripotent stem cells (PSC) provide a valuable tools in research as differentiation is rapid, cost effective and an extensive repertoire of transgenic lines contribute in our understanding of biology. However, protocols for directed differentiation of these cells has been inferior to their human counterparts. Here we demonstrate enhanced efficiency of neural differentiation by commencement from a naiive pluripotent ground-state, compared to a conventionally employed serum-dependent PSCs. Subsequently we establish novel protocols, involving early morphogen patterning of monolayer, feeder-free naïve PSC, resulting in the efficient generation of region-specific (dorsal forebrain, ventral forebrain, ventral midbrain and hindbrain) progenitors and neurons. These resultant populations will be valuble in understanding neural development, as well as disorders affecting specific neuronal populations.

## Discussion

Previous studies have described the generation of region-specific neural subtypes from Serum/LIF-dependent mESCs^2,3,5,38^. While these protocols have provided means to study neural development and transplantation therapies, several limitations are associated with these protocols - namely the variability between differentiations and heterogeneity of derived cultures. Underlying these variables is the reliance on either stromal cell co-culture that introduces a variable milieu of patterning signals, the use of spontaneous embryoid bodies for uncontrolled default differentiation and/or failures to replicate developmental sequences appropriately.

By comparison, neural differentiation strategies for human PSC have advanced significantly, with numerous studies generating largely homogenous cultures of specific regional neural subtypes under rapid patterning methodologies^8–11^. While these human protocols are invaluable to the field, rodent counterparts nevertheless remain an important biological tool that in comparison possess malleable genomes and undergo rapid embryonic development. These traits facilitate cost and time effective investigations into mammalian development as well as intra-species cell transplantation studies.

We report here the development of a feeder-free directed neural differentiation system for mESCs and iPSCs along dorso-ventral and rostro-caudal axes that aligns mPSC differentiation closely with human PSC counterparts. By mimicking *in vitro* the rapid temporal signaling gradients of rodent development an array of neural lineages from the dorsal forebrain, ventral forebrain, ventral midbrain and hindbrain were formed within 14 days, Fig. 8K.

Furthermore, we describe for the first time the use of naïve mPSC as a starting material for neural differentiation, resulting in differentiated cultures of improved homogeneity and eliminated contaminant pluripotent cells (Fig. 1). Interestingly the employment of naïve mPSC required precise recapitulation of developmental sequences, involving the transition of naïve cells (reflective of pre-implantation) temporarily to a more primed (post-implantation) state, by exposure to LIF, prior to the initiation of differentiation. The use of naïve mPSC likely underlies the robust fate restriction reported here, as recent studies evaluating naïve and serum-dependent mESC have demonstrated that the former are intrinsically homogeneous at transcriptomic and epigenomic levels, while the later express a range of lineage-specific genes likely to interfere with differentiation^14,17^.

Additional to the use of naïve stem cells, early patterning of mPSC reflective of embryonic development was imperative for restricted neural fate specification. Smad inhibition at the onset of differentiation forced neural specification towards the default dorsal forebrain lineage, reflected by the generation of Pax6+, Tbr2+, Emx2+ NPCs, and mirroring human PSC protocols. Following initial patterning and a week of maturation in neurotrophic factors and notch inhibitors, to induce a transition to post-mitotic neurons, dorsal forebrain progenitors differentiated into mature neurons that expressed anticipated cortical layer markers, such as Tbr1 and Ctip2. Previous mPSC protocols have reported the use of small molecule antagonists of the Sonic Hedgehog pathway to promote cortical specification from serum/LIF-dependent mESCs^5,39^. Our study found Shh inhibition was not required as evidenced by limited expression of the ventral floor-plate marker Foxa2 cells within the cultures and enrichment of dorsal cortical markers. Initiating differentiation from naïve mESC, as opposed to S/L-dependent mESCs, may underlie the improved patterning outcomes, despite a reduction in administered extrinsic signals.

Conversely, Shh agonists (Purmorphamine and recombinant Shh) were required from day 1 of differentiation to ventralize NPCs, resulting in the acquistation of a hypothalamic identity, as seen by expression of *Nhlh2* and *Otp* ^40^. Of note, ventralized NPCs did not express ganglionic eminence markers such as Nkx2.1 and Olig2, suggesting that the Shh and puromorphomine concentrations used maximally activated the Shh pathway to produce the most ventral structures of the developing rostral neural tube. Following maturation, ventral forebrain NPCs developed into GABAergic neurons, a population present in the adult hypothalamus, in addition to FoxA2-TH+ dopaminergic neurons^40,41^. Interstingly, the addition of early Wnt inhibition, to reduce the extent of floorplate ventralization of the progenitors, we were able to direct cells to adopt a GE identity. These GE-like progenitors were positive for both Nkx2.1 and Olig2 and continued to develop into GABAergic neurons, a neuronal population that arises in the GE during ventral telencephalic development.

Differentiation to the ventral midbrain was also acheived from naïve mPSCs with the addition of caudalization cues supplied by FGF8 and the GSK3b inhibitor CHIR9902. This sequence of immediate neuralization, ventralization and rostrocaudal positioning closely mirrors hPSC protocols for VM differentiation^8,10,21^. Post-mitotic neurons were obtained rapidly, within 14 days of differentiation, and confirmed to be *bona fide* ventral midbrain dopamine neurons based on Lmx1a and Pitx3 expression (using our knock-in reporter line) as well as FoxA2, in addition to gene transcripts for *En1*, *Nurr1* and *Lmx1a*.

Finally, ventral hindbrain specification was attained by increased caudalisation of NPCs, using amplified canonical Wnt activation. Resultant NPCs showed evidence of hindbrain identity based upon the presence of Zic1 and lack of Otx2 immunoreactivity in conjunction with motor (Islet1) and serotonergic (5-HT) neuronal markers^36,37^.

Demonstrative of the efficiency of these new protocols was the relative lack of off-target cells within each populations. Genes known from *in vivo* neural development to be to be expressed within select progenitor/neuronal populations were largely restricted within our cultures, such that dorsal identity gene *Pax6* was only expressed in dorsal forebrain cultures, yet rhombencephalic genes, *Hoxa1* and *Zic1*, were only expressed in hindbrain cultures. While the combination of restricted transcriptional gene expression as well as protein expression (and often protein co-localisation) enabled confirmation of the presence of restricted neuronal populations in some instances, we recognize that the efficiency of these differentiation protocols still contains variability, and one that is higher than comparative human PSC protocols. At one extreme >75% of naïve mESC, differentiated along a VM lineage, adopted a VM DA fate (quantitatively confirmed by TH+ Pitx3+ colocalisation) yet VF cultures were decidedly more heterogeneous (including both GABA+, TH+ FoxA2− and TH+ FoxA2+ neurons), proving challenging for defintive confirmation of neuronal identity from adjacent populations. We speculate that this, in part, is a likely consequence of the rapid differentiation of mouse PSCs compared to human counterparts that allows less time to orchestrate regional patterning and terminal signals. As such, more extensive transcriptional, histochemical, biochemical and functional/electrophysiological profiling studies will be required in order to confirm the true efficiency of each of these described protocols.

In summary, using naïve mPSC, we describe the robust regional specification of PSC through the early manipulation of rostro-caudal and dorso-ventral morphogen gradients. These protocols, and resultant neuronal populations, may provide important tools for understanding mammalian neural development, aide in our understanding of brain-related disorders and assist in the development of new targeted therapies including cell replacement therapy.

## Electronic supplementary material

Supplementary Figures
