# Supplementary material for: Specification of murine ground state pluripotent stem cells to regional neuronal populations
Source: Sci Rep. 2017 Nov 22;7:16001. doi: 10.1038/s41598-017-16248-x (PMC5700195; doi:10.1038/s41598-017-16248-x)
Supplement: Supplementary file 1 — Supplementary Figures [file 41598_2017_16248_MOESM1_ESM.pdf]

Supplementary Text:

Specification of murine ground state pluripotent stem cells to regional neuronal populations

Walaa F. Alsanie[1], Jonathan C. Niclis[1], Cameron P. Hunt[1,2], Vanessa Penna[1], Isabelle R. De Luzy[1], Christopher R. Bye[1], Colin W. Pouton[2], John Haynes[2], Jaber Firas[3], Lachlan H. Thompson[1], Clare L. Parish[1]

[1]The Florey Institute of Neuroscience and Mental Health, The University of Melbourne, Melbourne, Australia. [2]Monash Institute of Pharmaceutical Sciences and, [3]The Australian Regenerative Medicine Institute, Monash University, Melbourne, Australia.

Correspondence: C.L. Parish: clare.parish@florey.edu.au

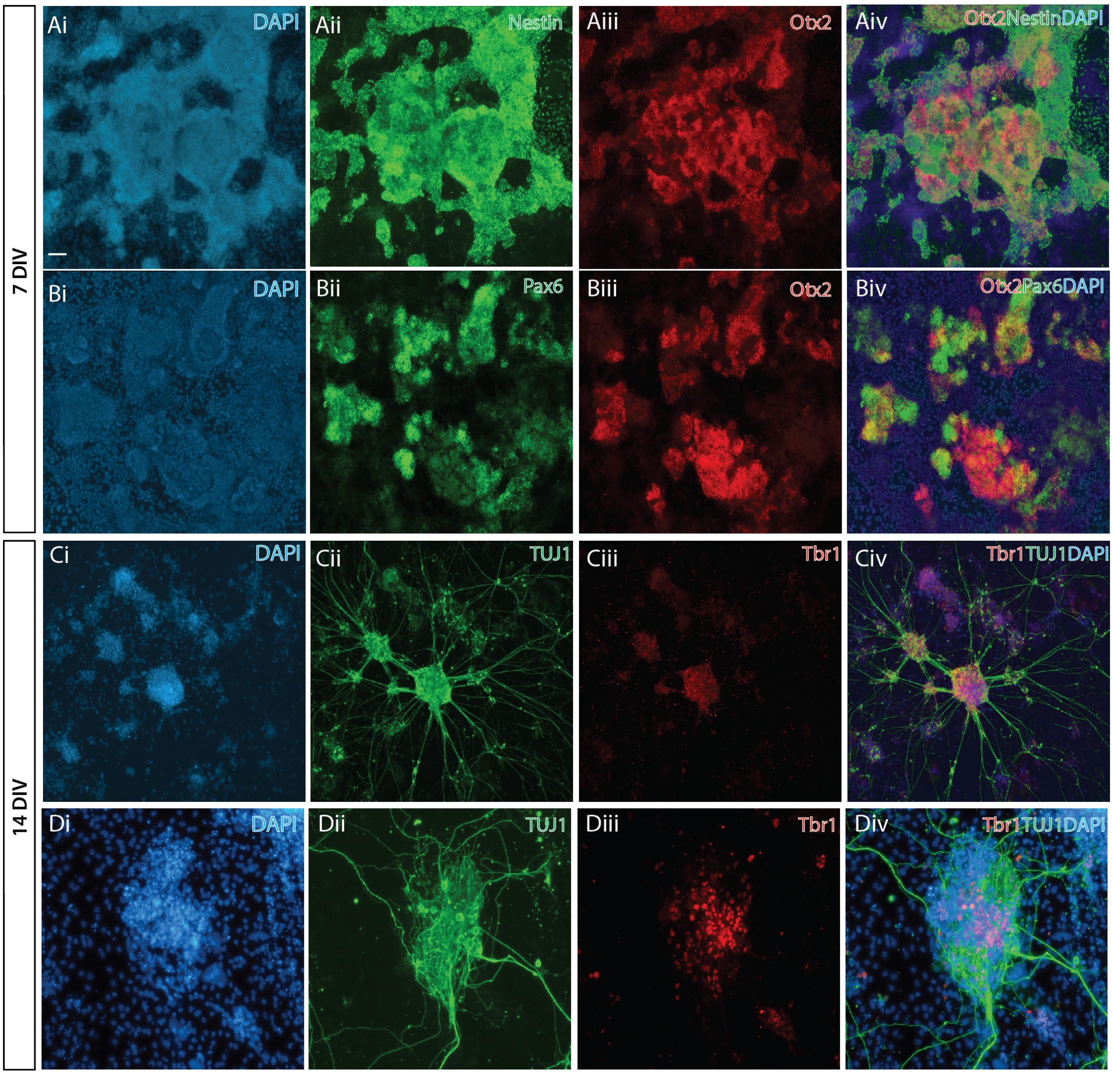

Supplementary Figure 1: Ground state mouse iPSC differentiate into dorsal forebrain progenitors and neurons. (A,B) Representative micrographs depicting expression of Nestin, Otx2 and Pax6 in dorsal forebrain progenitors at day7. (C) Mature dorsal forebrain neurons expressing TUJ1 and Tbr1 at day14. Images show culture overviews, while inserts show immunocytochemical labeling at the resolution of individual cells. Scale = 100 um.

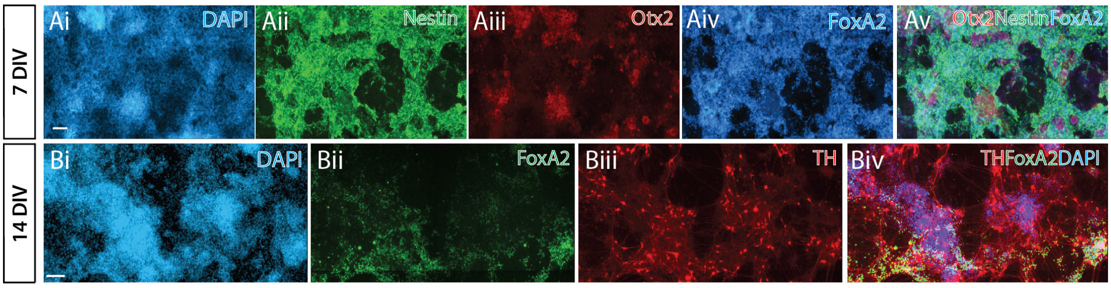

Supplementary Figure 2: Ground state mouse iPSC differentiate into ventral forebrain progenitors and mature neurons. (A) Representative micrographs depicting expression of Nestin, Otx2 and FoxA2 in ventral forebrain progenitors at day7. (B) Analysis at day 14 revealed that neurons expressed TH but not FoxA2, indicative of ventral forebrain, and not midbrain, dopaminergic neurons. Scale = 100 um. (n=3).

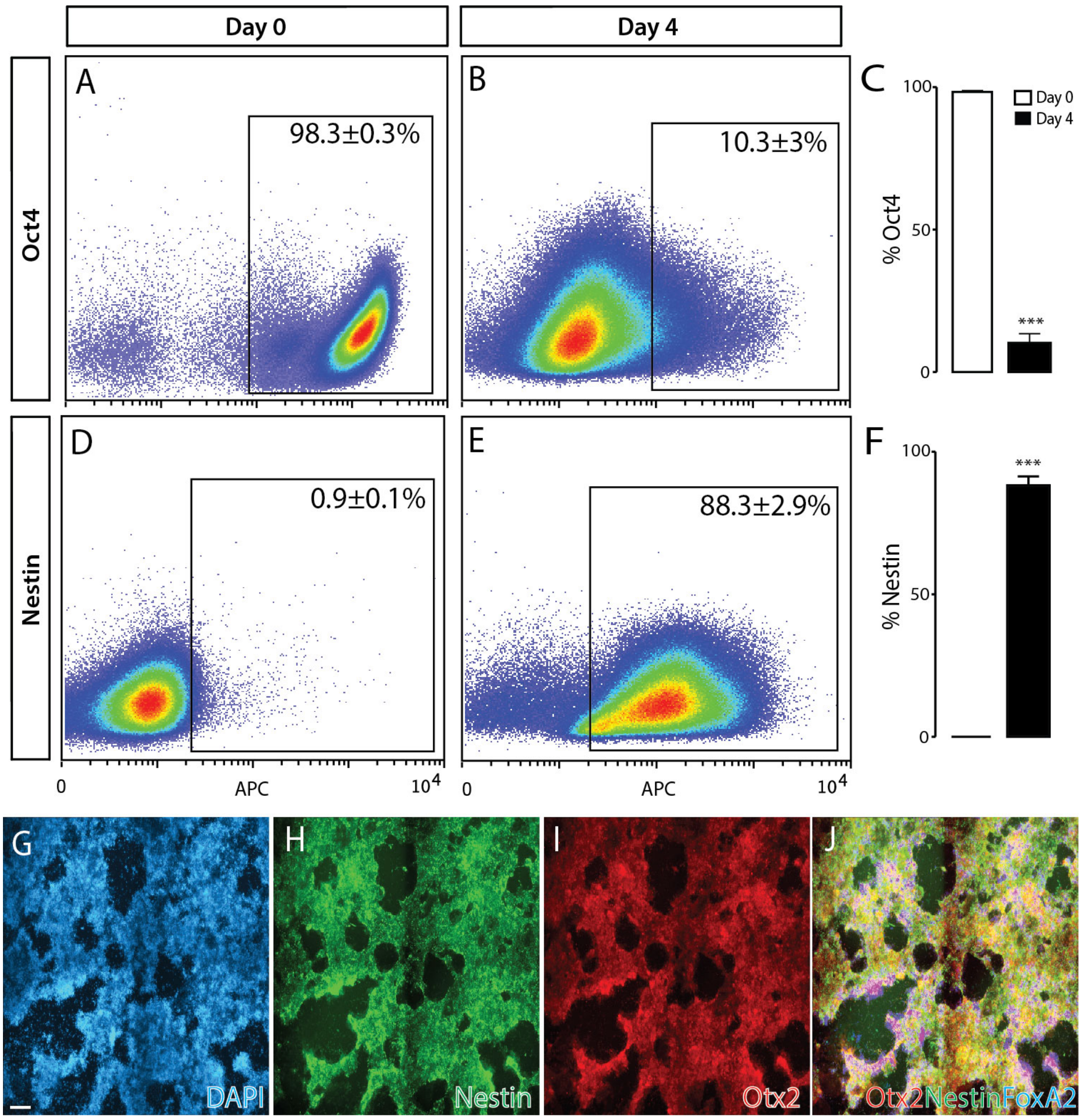

Supplementary Figure 3: Ground state mESC differentiate into Otx2-positive neural progenitors after 4days of differentiation. (A,B,D,E) Flow cytometry plots and (C,F) graphs show naïve mESCs that, upon early neuronal specification, results in downregulation of Oct4 and the generation of a high yields of Nestin-positive progenitors. (G-J) Representative images showing wide expression of Nestin and Otx2 at day 4, indicative of the derivation of mid-forebrain neural progenitors. (G'-J') Enlarged images show the co-expression of Nestin and Otx2 in neural progenitors. Data (n=3) represented as mean ± SEM, Students t-test. ***$p<0.0001$. Scale = 100 um.

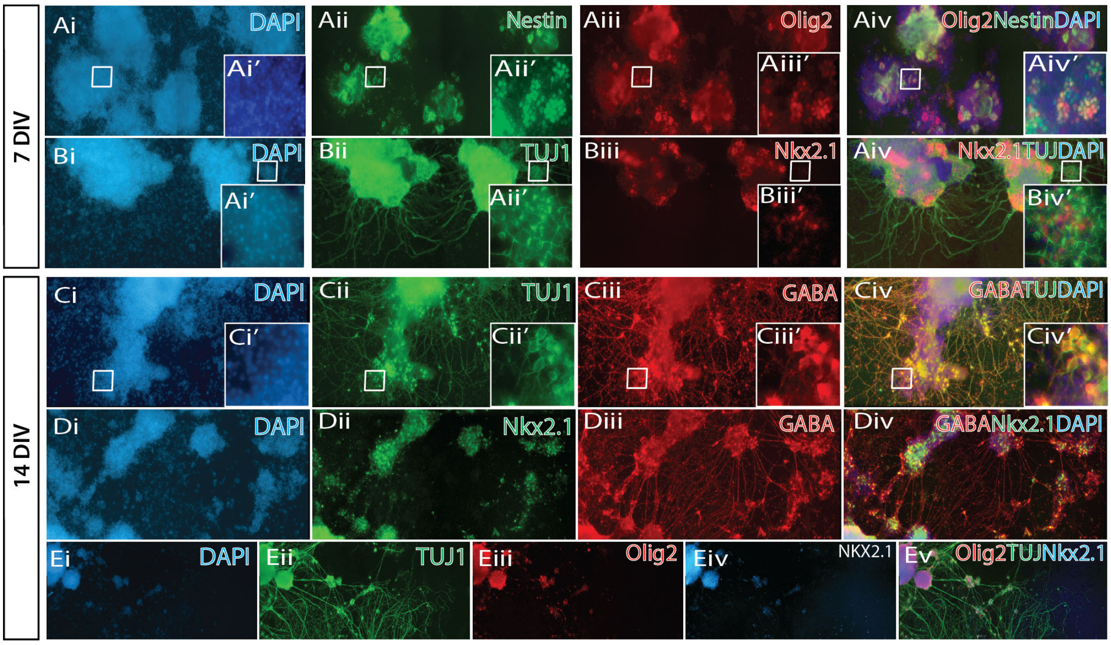

Supplementary Figure 4: Ground state mouse iPSC differentiate into GE-specific progenitors and mature neurons. (A,B) Representative images highlighting the expression of Nkx2.1, Olig2 and TUJ1 in mIPSC-derived GE progenitors. (C) By day 14, these progenitors differentiated in TUJ+, GABA+ neurons, with (D) many cells co-expressing Nkx2.1, as well as (E) Olig2, indicative of MGE interneurons. Images show culture overviews, while inserts show immunocytochemical labeling at the resolution of individual cells. Scale= 100 um.

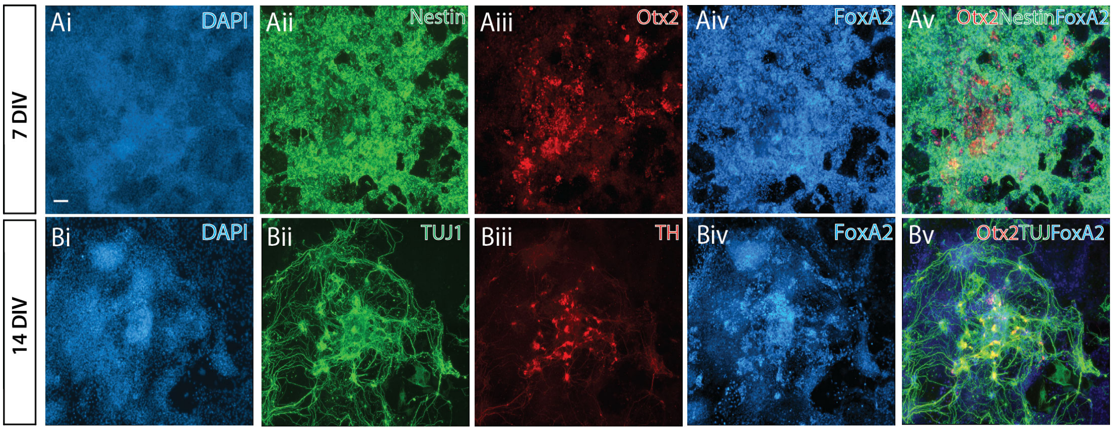

Supplementary Figure 5: Ground state mouse iPSC differentiate into ventral midbrain progenitors and mature neurons. (A) Representative images highlighting the expression of Nestin, Otx2 and FoxA2 in neural progenitors at day7. (B) By day 14, progenitors matured into TUJ+ neurons that co-expressed TH+ and FoxA2+, indicative of ventral midbrain dopamine neurons. Images show culture overviews, while inserts show immunocytochemical labeling at the resolution of individual cells. Scale = 100 um.

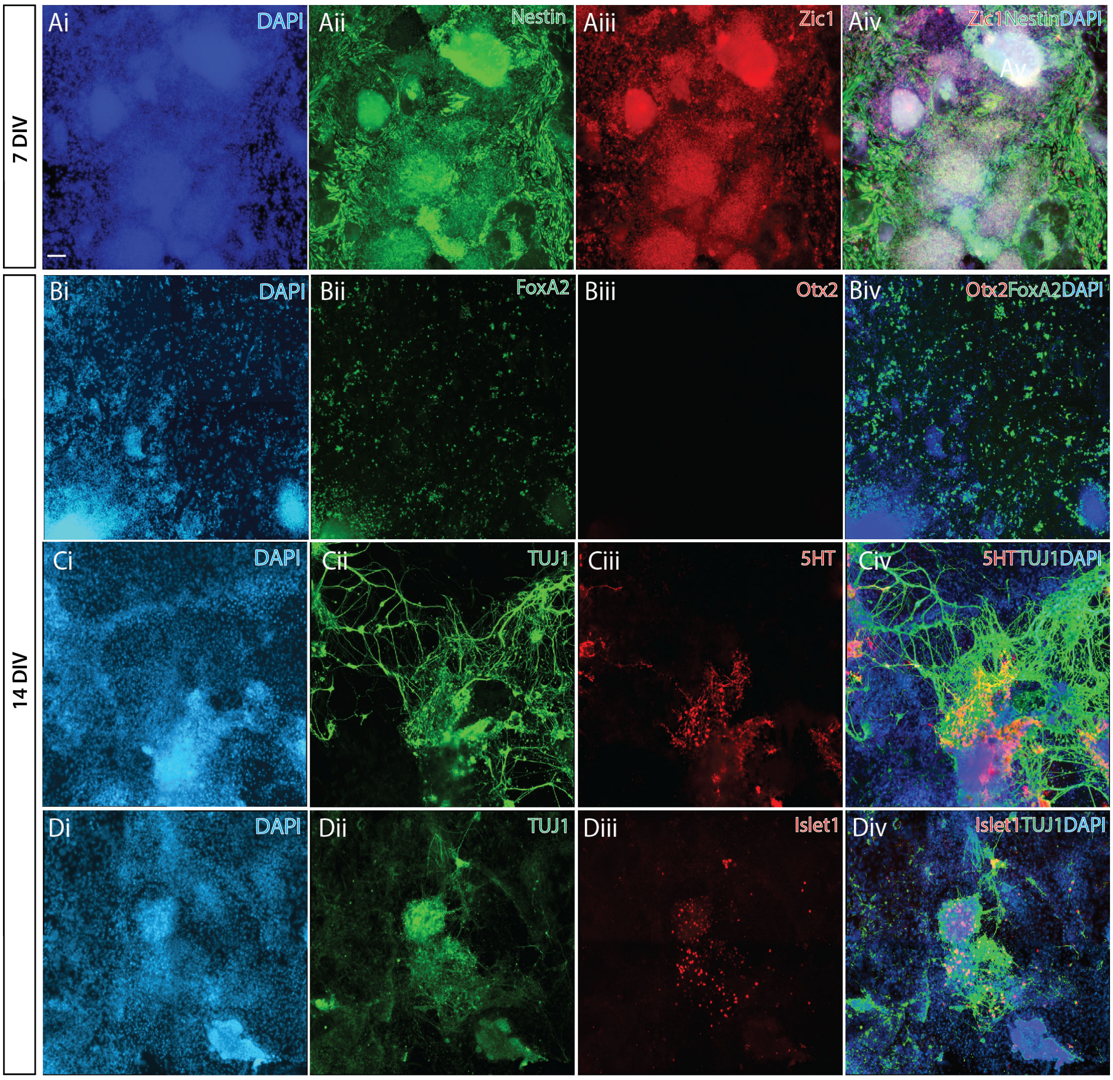

Supplementary Figure 6: Ground state mouse iPSC differentiate into hindbrain progenitors and mature neurons. (A) Representative images highlighting the expression of Nestin and Zic1 in hindbrain progenitors at day7. At day 14, appropriately patterned ventral hindbrain neurons were confirmed by the absence of Otx2 and expression of FoxA2. Subpopulations of (C) 5HT+/TUJ+ and (D) Islet1+/TUJ+ neurons could be identified, indicative of hindbrain serotonergic and motor neurons, respectively. Images show culture overviews, while inserts show immunocytochemical labeling at the resolution of individual cells. Scale = 100 um.
